# COVID-19 Vaccine Booster Dose Acceptance: Systematic Review and Meta-Analysis

**DOI:** 10.3390/tropicalmed7100298

**Published:** 2022-10-13

**Authors:** Shaimaa Abdelaziz Abdelmoneim, Malik Sallam, Dina Mohamed Hafez, Ehab Elrewany, Hesham Metwalli Mousli, Elsayed Mohamed Hammad, Sally Waheed Elkhadry, Mohammed Fathelrahman Adam, Amr Abdelraouf Ghobashy, Manal Naguib, Ahmed El-Sayed Nour El-Deen, Narjiss Aji, Ramy Mohamed Ghazy

**Affiliations:** 1 Clinical Research Administration, Alexandria Directorate of Health Affairs, Egyptian Ministry of Health and Population, Alexandria 21554, Egypt; 2 Department of Pathology, Microbiology and Forensic Medicine, School of Medicine, The University of Jordan, Amman 11942, Jordan; 3 Department of Clinical Laboratories and Forensic Medicine, Jordan University Hospital, Amman 11942, Jordan; 4 Pharmacy Department, Alexandria University Students Hospital, Alexandria 5422023, Egypt; 5 Tropical Health Department, High Institute of Public Health, Alexandria University, Alexandria 21561, Egypt; 6 Continuous Quality Improvement & Patient Safety Department, Alexandria Urology Hospital, Alexandria 5442045, Egypt; 7 Faculty of Medicine, Alexandria University, Alexandria 21544, Egypt; 8 Department of Epidemiology and Preventive Medicine, National Liver Institute, Menoufia University, Menoufia 32511, Egypt; 9 Faculty of Pharmacy, University of Science and Technology, Khartoum 12810, Sudan; 10 Faculty of Medicine, Kafrelsheikh University Hospital, Kafr el-Sheikh 33516, Egypt; 11 Egyptian Ministry of Health and Population, Alexandria 21554, Egypt; 12 Department of Physiology, Faculty of Medicine, Al-Azhar University, Assiut 71524, Egypt; 13 Faculty of Medicine and Pharmacy of Rabat, Mohammed V University, Rabat 10100, Morocco

**Keywords:** vaccine resistance, vaccine rejection, vaccine hesitancy, vaccine preference, vaccine attitude, secondary immunization, public health practice, communicable disease control

## Abstract

The World Health Organization (WHO) recommended coronavirus disease 2019 (COVID-19) booster dose vaccination after completing the primary vaccination series for individuals ≥18 years and most-at-risk populations. This study aimed to estimate the pooled proportion of COVID-19 vaccine booster dose uptake and intention to get the booster dose among general populations and healthcare workers (HCWs). We searched PsycINFO, Scopus, EBSCO, MEDLINE Central/PubMed, ProQuest, SciELO, SAGE, Web of Science, Google Scholar, and ScienceDirect according to PRISMA guidelines. From a total of 1079 screened records, 50 studies were extracted. Meta-analysis was conducted using 48 high-quality studies according to the Newcastle-Ottawa Scale quality assessment tool. Using the 48 included studies, the pooled proportion of COVID-19 vaccine booster dose acceptance among 198,831 subjects was 81% (95% confidence interval (CI): 75–85%, *I*^2^ = 100%). The actual uptake of the booster dose in eight studies involving 12,995 subjects was 31% (95% CI: 19–46%, *I*^2^ = 100%), while the intention to have the booster dose of the vaccine was 79% (95% CI: 72–85%, *I*^2^ = 100%). The acceptance of the booster dose of COVID-19 vaccines among HCWs was 66% (95% CI: 58–74%), *I*^2^ = 99%). Meta-regression revealed that previous COVID-19 infection was associated with a lower intention to have the booster dose. Conversely, previous COVID-19 infection was associated with a significantly higher level of booster dose actual uptake. The pooled booster dose acceptance in the WHO region of the Americas, which did not include any actual vaccination, was 77% (95% CI: 66–85%, *I*^2^ = 100%). The pooled acceptance of the booster dose in the Western Pacific was 89% (95% CI: 84–92%, *I*^2^ = 100), followed by the European region: 86% (95% CI: 81–90%, *I^2^* = 99%), the Eastern Mediterranean region: 59% (95% CI: 46–71%, *I*^2^ = 99%), and the Southeast Asian region: 52% (95% CI: 43–61%, *I*^2^ = 95). Having chronic disease and trust in the vaccine effectiveness were the significant predictors of booster dose COVID-19 vaccine acceptance. The global acceptance rate of COVID-19 booster vaccine is high, but the rates vary by region. To achieve herd immunity for the disease, a high level of vaccination acceptance is required. Intensive vaccination campaigns and programs are still needed around the world to raise public awareness regarding the importance of accepting COVID-19 vaccines needed for proper control of the pandemic.

## 1. Introduction

Coronavirus disease 2019 (COVID-19) is a highly communicable infectious disease caused by severe acute respiratory syndrome coronavirus 2 (SARS-CoV-2) [1]. About 3 years have passed since its first reporting in Wuhan, China; still, SARS-CoV-2 continues to pose serious threats to the global health [1,2]. Based on the World Health Organization (WHO) statistics, the burden of COVID-19 is manifested in more than 600 million confirmed cases and 6.48 million deaths worldwide as of 7 September 2022, with different patterns and predictors of morbidity and mortality [3,4,5]. As a result, effective preventive measures were warranted with intensive and swift efforts directed towards the development of effective COVID-19 vaccines [6]. By early September 2022, 47 COVID-19 vaccines have been approved by at least one country, and the WHO granted emergency use listing (EUL) to 12 vaccines [7].

Despite the timely development of effective and safe COVID-19 vaccines, hesitancy to get vaccinated emerged as a major hindrance to preventive efforts [8,9,10]. In addition, waning immunity following infection or vaccination and the continuous emergence of SARS-CoV-2 variants with immune escape potential underlined the necessity of booster doses of COVID-19 vaccines [11,12,13]. Booster dose can be defined as an extra dose of vaccine administered following the completion of a primary vaccination series [14]. In the context of COVID-19 vaccination, it is recommended to take the booster dose if it is available based on current evidence showing that protective immunity wanes 4–6 months following the primary vaccination [11,15,16]. Receiving a booster dose of COVID-19 vaccines has been proved to significantly increase immunogenicity and to improve the peak antibody levels following the primary immunization series among healthy adults [17,18,19].

Currently, the WHO recommends that individuals aged 18 years or older have a booster dose of COVID-19 vaccines 4–6 months following the completion of the primary vaccination series [20]. As of 8 September 2022, data on the uptake of COVID-19 vaccines point to approximately 4 billion people who are fully vaccinated, 4.8 billion who received at least one dose of the vaccine, and only 749 million people who received a booster dose of COVID-19 vaccines [21]. Therefore, the investigation of reasons behind hesitancy to have booster doses of COVID-19 vaccination is warranted, which can help to understand the determinants of vaccine uptake, which in turn can help in designing well-informed vaccination campaigns and strategies to promote vaccination [22,23,24].

It has been shown that the prevalence of the behavioral intention to receive a COVID-19 booster dose among the general population is variable in different countries with a range of 62–67% in the U.S., 67–71% in Poland, and 94% in China [25,26,27]. Multiple factors are associated with the acceptance of booster doses of COVID-19 vaccination, including high levels of fear of COVID-19 (low complacency) and trust in COVID-19 vaccination (high confidence), as well as low levels of fear of a booster dose or a new COVID-19 vaccine [28].

The current systematic review and meta-analysis aimed to address the acceptance of the general population of the booster dose of COVID-19 vaccination and to identify its associated determinants. Through identifying the main predictors of booster dose vaccine acceptance, public health authorities could be able to increase the acceptance and uptake rates of booster doses, resulting in higher vaccination coverage and population immunity with proper control of the ongoing COVID-19 pandemic.

## 2. Materials and Methods

### 2.1. Study Measures

The primary study measure was the estimation of the pooled proportion of COVID-19 vaccine booster dose acceptance and actual uptake. Acceptance of the booster dose of COVID-19 vaccination was defined as the willingness to receive the vaccine as opposed to vaccine reluctance or rejection.

The secondary study measures included: (1) identification of the determinants of COVID-19 vaccine booster dose acceptance; (2) assessment of COVID-19 vaccine booster dose vaccine acceptance among healthcare workers (HCWs); and (3) evaluation of the differences in COVID-19 booster dose vaccine acceptance across different WHO regions.

### 2.2. Data Sources

This meta-analysis was guided by the 2020 Cochrane Handbook of Systematic Review and Meta-Analysis, with respect to the preferred reporting items of the systematic review and meta-analysis (PRISMA) checklist [29,30]. To access the acceptance and coverage of COVID-19 vaccine booster doses, the search process started on 28 May 2022 and conducted on 4 June 2022 for studies that had been published up until 4 June 2022. In addition to grey literature, published studies in the following databases were included: PsycINFO, Scopus, EBSCO, MEDLINE Central/PubMed, ProQuest, SciELO, SAGE, Web of Science, Google Scholar, and ScienceDirect. Search terms were determined and approved after the consultation of the PubMed help desk. The used keywords are presented in Table S1.

### 2.3. Data Extraction and Study Selection

All studies reporting the acceptance of a COVID-19 vaccine booster dose were included with no language restriction or vaccine-type restriction. Abstract-only papers, proposals, conference proceedings, editorials, author responses, reviews, case reports, case series, books, and duplicate records were excluded.

**Figure 1:**
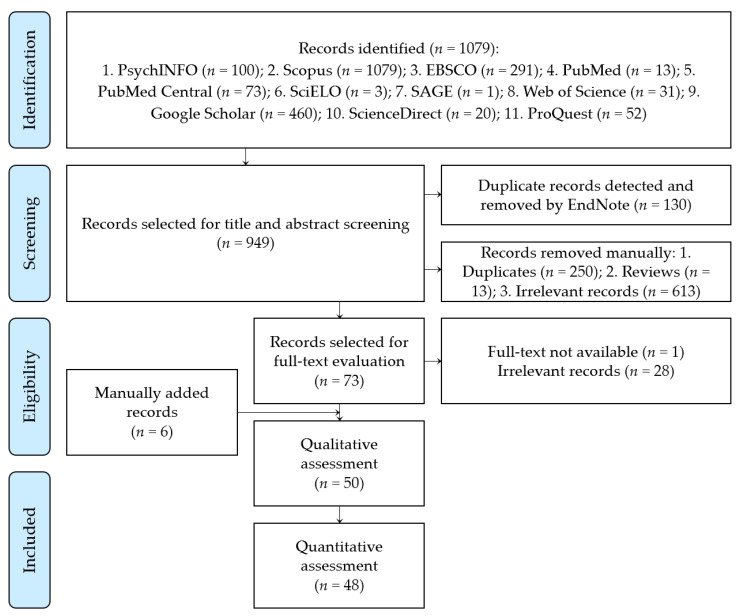
PRISMA flow chart of the included studies.

The PRISMA flow chart for the different steps of the current meta-analysis is depicted in Figure 1. All articles were imported into EndNote X8 for Windows (Thomson ResearchSoft, Stanford, CA, USA), to detect and remove duplicates. After the citation’s exportation to an MS Excel sheet containing the authors’ names, publication year, journal name, digital object identifier (DOI), URL link, and abstract, the authors screened both the title and the abstract. This was followed by full-text screening to identify the eligible articles. Screening was performed independently by four authors (S.A.A., H.M.M., E.M.H., and A.E.-S.N.E.-D). The senior author (R.M.G.) solved any disagreement. Further manual search for eligible citations was conducted through careful examination of the references of the included studies and studies citing the selected articles using PubMed and Google Scholar. All included articles were extracted to an MS Excel sheet with the following predefined data: publication year, authors’ names, country, study design, study setting, study population, sample size, duration of the study, inclusion and exclusion criteria, vaccine booster dose acceptance, predictors of booster dose vaccine acceptance, situation of participants regarding full COVID-19 vaccination, and the assessment tool used. Supplementary files were reviewed for any relevant information. The review protocol was registered at PROSPERO (registration: CRD42022333758), and the MS Excel sheets including the relevant used data are available online using the following link: https://docs.google.com/spreadsheets/d/1PyffvdDMqXJuzgy4T6WSIl3qJ1Uyf748/edit?usp=sharing&ouid=104751321858570795359&rtpof=true&sd=true (accessed on 9 October 2022) [31].

### 2.4. Investigations of Heterogeneity

Cochrane’s Q test (*I*^2^) was used to assess and measure heterogeneity between studies [29]. Due to substantial heterogeneity, DerSimonian and Laird random-effects models were applied to the pooled outcomes. The degree of heterogeneity was classified into:

- 0% to 40%: might not be important;
- 30% to 60%: may represent moderate heterogeneity;
- 50% to 90%: may represent substantial heterogeneity;
- 75% to 100%: considerable heterogeneity.

### 2.5. Publication Bias

Publications’ bias was assessed by visual inspection of the funnel plot and statistically by Egger’s regression test [29].

### 2.6. Quality Assessment

Quality assessment (QA) was based on the Newcastle-Ottawa Scale quality assessment tool customized for cross-sectional and cohort studies [32]. The quality of studies was either very good (9–10 points), good (7–8 points), satisfactory (5–6 points), or unsatisfactory (0–4 points) [33]. The assessment was performed by two independent reviewers (S.W.E. and E.E.) and further checked by two additional reviewers (S.A.A. and M.F.A.).

### 2.7. Statistical Analysis

The statistical analyses were conducted using the R 4.2.1 software (R Foundation for Statistical Computing, Vienna, Austria). Due to the heterogeneity between the studies, a random effect model was used for illustrating the pooled proportion of booster dose COVID-19 vaccine acceptance. To explain the statistical heterogeneity between the included studies, meta-regression analysis was conducted. Sensitivity analysis was performed using leave-one-out analysis to identify the influential studies and to recalculate the pooled proportion a number of times, removing an influential study at each time (Figure S1).

Subgroup analysis was conducted for the assessment of total COVID-19 booster dose vaccine acceptance among different WHO regions and among HCWs based on the intention to get a booster dose of the vaccine and the actual booster dose vaccination uptake (Figure S1).

## 3. Results

The primary search using the aforementioned databases identified 1079 records, from which 949 studies were screened using the title and abstract, after excluding 130 duplicates by the EndNote X8 software. We excluded 613 irrelevant studies (those that did not report COVID-19 vaccine booster dose acceptance or uptake), 13 review articles, and 250 duplicates that were detected manually during title and abstract screening. After full-text screening, 1 unavailable citation was excluded, and 28 articles were irrelevant. Then, 44 articles were eligible for data extraction in addition to 6 records found through manual search and track citations. After QA, 2 studies were excluded because of their unsatisfactory score [34,35]. Finally, 48 studies were eligible for meta-analysis (Figure 1).

### 3.1. Characteristics of the Included Studies

Out of the 50 included studies, 32 were published in 2022, while 18 were published in 2021. The total number of participants among the 50 included studies was 194,410 subjects from 23 different counties across 6 WHO regions, with only 1 study conducted across 2 different regions [36]. Most studies were cross-sectional except 4 longitudinal survey studies [37,38,39,40] and 2 retrospective cohort studies [41,42]. The total number of HCWs in the included studies was 9125 subjects. The included studies are overviewed in Table 1 and Table 2. The quality of the included studies ranged between very good and unsatisfactory according to the assessment tool as follows: 3 studies were classified as very good studies, 10 were classified as good studies, 35 were classified as satisfactory studies, and 2 studies were classified as unsatisfactory [34,35].

### 3.2. Risk of Publication Bias

**Figure 2:**
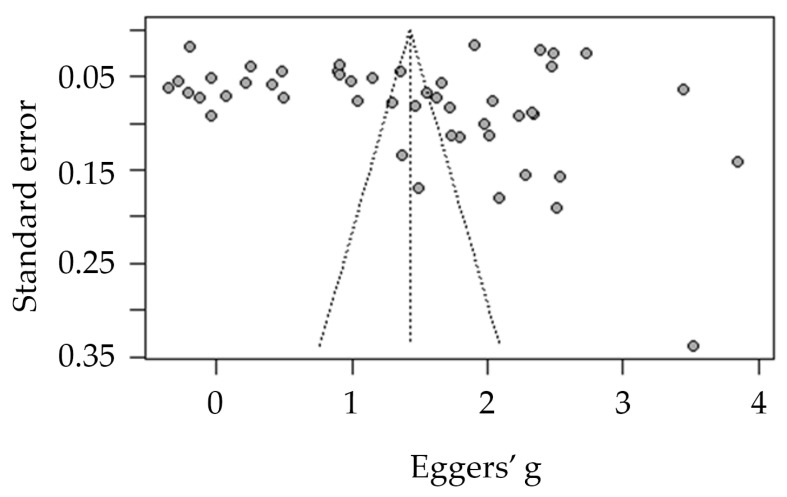
Funnel plot of publication bias of the included studies.

The absence of publication bias was evident by a nonsignificant Eggers’ test t = −0.41 (95% CI: −2.47 to −14.08, *p* = 0.670) and the funnel plot, which did not indicate the presence of funnel plot asymmetry (Figure 2).

### 3.3. Proportion of COVID-19 Acceptance

#### 3.3.1. Actual and Intentional Acceptance of COVID-19 Booster Dose Vaccination

**Figure 3:**
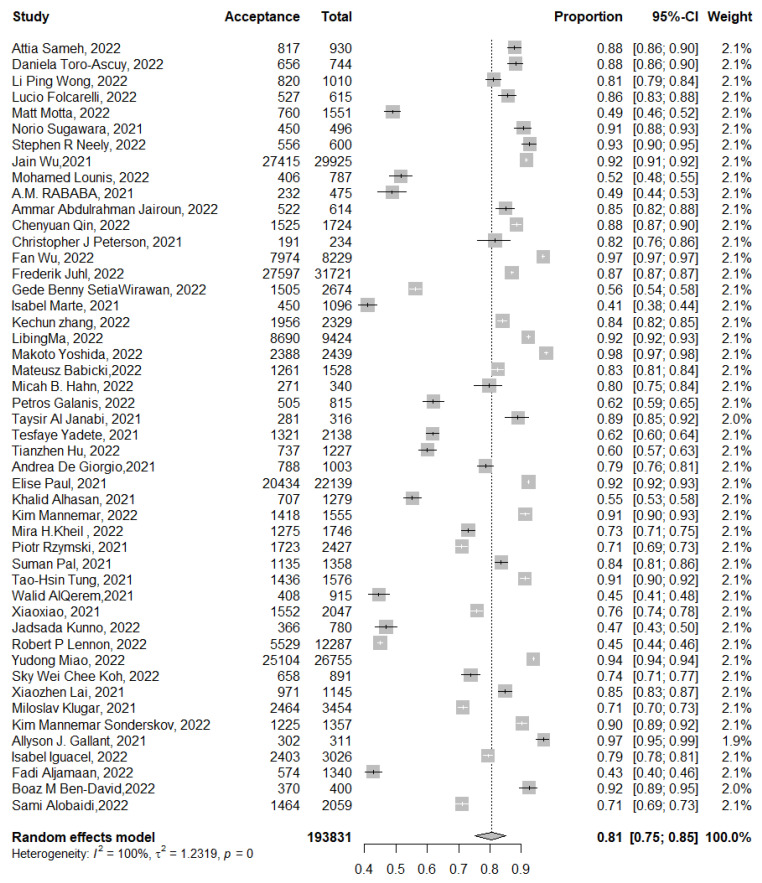
The pooled proportion of total COVID-19 vaccine booster dose acceptance (actual uptake and intention to take the booster dose. *p* = 0 denoted a *p*-value < 0.001.

The pooled proportion of COVID-19 vaccine booster dose acceptance for the eligible 48 studies with 193,831 participants was 81% (95% CI: 75–85%, *I*^2^ = 100%). The highest acceptance rate was 98% (95% CI: 97–98%) [41], while the lowest proportion of acceptance was 41% (95% CI: 38–44%) [46] (Figure 3). Subgroup analysis according to the actual vaccination or intention to be vaccinated did not explain this heterogeneity (Supplementary Figure S1). Since six studies represented the COVID-19 vaccine booster dose acceptance as actual vaccination or intention to be vaccinated [51,54,55,61,66,73], it was a necessary to identify the pooled proportion for the actual booster dose vaccination and intention to receive the booster dose separately.

#### 3.3.2. Actual Uptake of COVID-19 Vaccine Booster Dose

**Figure 4:**
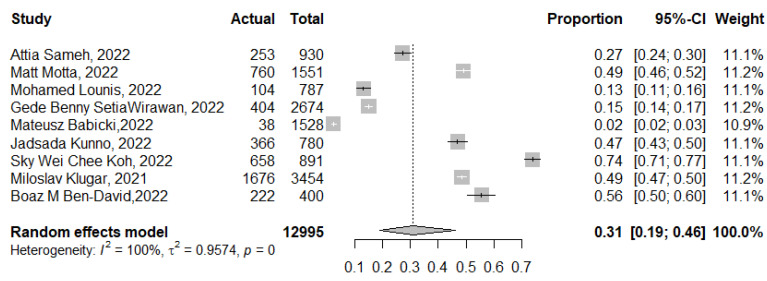
Forest plot of the pooled proportion of COVID-19 vaccine booster dose actual uptake. *p* = 0 denoted a *p*-value < 0.001.

The attitude of 12,995 participants included in 8 studies was analyzed. The pooled proportion of the actual uptake of a COVID-19 vaccine booster dose was 31% (95% CI: 19–46%, *I*^2^ = 100%), ranging from 2% (95% CI: 2–3%) [51] to 74% (95% CI: 71–77%) [56] (Figure 4). After excluding the multicollinearity by correlation and the variance inflation factor (VIF), meta-regression succeeded in explaining 51% of this high heterogeneity with residual heterogeneity τ^2^ = 0.46 (SE = 0.48). The most fitted model revealed that previous COVID-19 infection increases the actual booster dose acceptance significantly: 0.002 (95% CI: −0.000 to 0.003, *p* = 0.040); being employed: −2.27 (95% CI: −3.46 to −1.08, *p* < 0.001); vaccine type: −2 (95% CI: −3.50 to −0.50, *p* = 0.008); and large sample size greater than 1000 having a significant negative effect on the actual vaccination: −1.49 (95% CI: −2.43 to −0.54, *p* = 0.002). Being HCWs and the study setting in a high-income country had no significant effect on the uptake of the booster dose: −0.31 (95% CI: −0.97 to 0.34, *p* = 0.350) and 1.28 (95% CI: −0.169 to 2.730, *p* = 0.083), respectively.

#### 3.3.3. Intentional Acceptance of COVID-19 Vaccine Booster Dose

**Figure 5:**
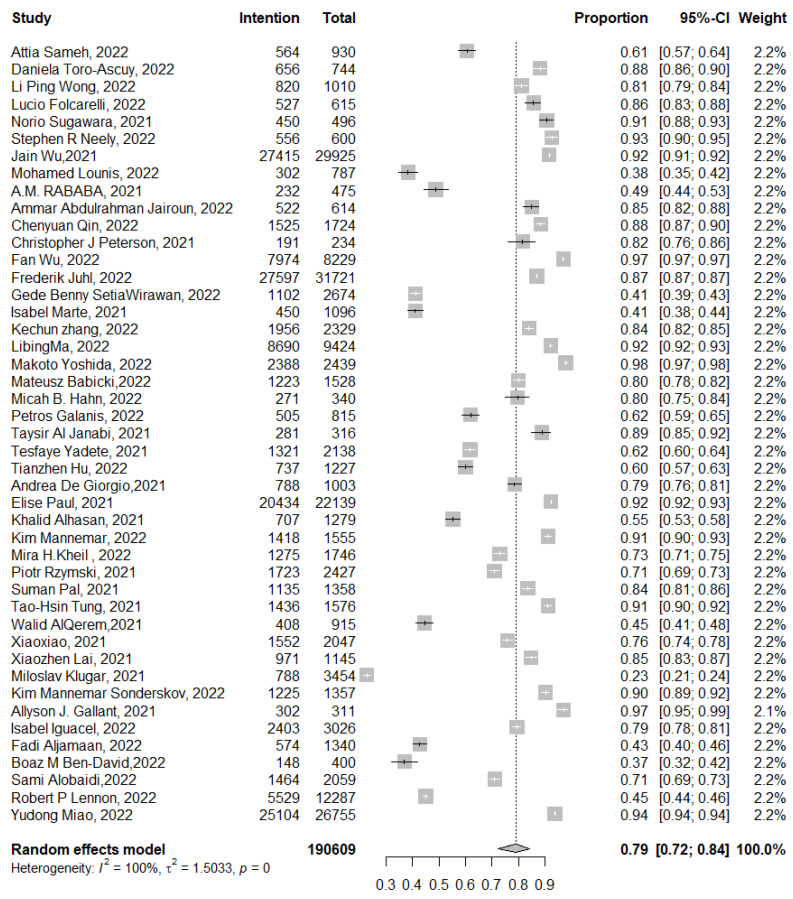
Forest plot of the pooled proportion of intentional COVID-19 booster dose acceptance. *p* = 0 denoted a *p*-value < 0.001.

Of the 190,609 participants included in 45 studies, the pooled proportion of intentional booster dose vaccine acceptance was 79% (95% CI: 72–84%, *I*^2^= 100%), ranging between 23% (95% CI: 21–24%) [55] and 97% (95% CI: 95–99%) [37] (Figure 5). Meta-regression for the studies that addressed intentional booster dose vaccination explained 52% of this heterogeneity with residual heterogeneity τ^2^ = 0.749 (SE = 0.293). The most fitted model revealed that previous COVID-19 infection decreased the intention for booster dose: −0.001 (95% CI: −0.002 to −0.000, *p* = 0.034); being employed increased the intention for booster dose vaccine acceptance: 0.800 (95% CI: 0.14–1.45, *p* = 0.016); being HCWs had no significant effect on the intention for booster dose: 0.018 (95% CI: −0.37 to 0.41, *p* = 0.920); and the study setting in the Western Pacific region increased the intention to receive the vaccine booster dose: 2.23 (95% CI: 0.277–4.187, *p* = 0.025); nevertheless, the studies conducted in the Americas, Europe, Eastern Mediterranean region, or Southeast Asia did not show such an effect: 1.25 (95% CI: −0.642 to 3.1560, *p* = 0.194), 1.52 (95% CI: −0.38 to 3.44, *p* = 0.110), 0.86 (95% CI: −1.11 to 2.84, *p* = 0.390), and 0.35 (95% CI: −2.30 to 3.01, *p* = 0.790), respectively.

### 3.4. COVID-19 Booster Dose Vaccine Acceptance among HCWs

**Figure 6:**
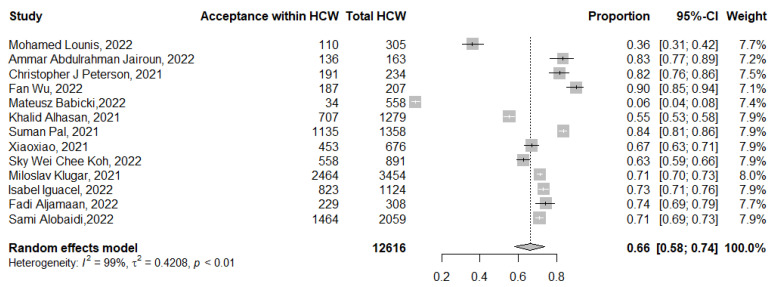
Acceptance of COVID-19 booster dose vaccination (actual and intentional) among healthcare workers (HCWs).

Among the 15 studies that included HCW participants, 13,420 HCWs were asked about their attitude towards booster dose, but only 13 studies with 12,616 HCWs reported the acceptance proportion among HCWs. The pooled proportion of COVID-19 vaccine booster dose acceptance among HCWs was 66% (95% CI: 58–74%, *I*^2^ = 99%) ranging from 36% (95% CI: 31–42%) [54] to 90% (95% CI: 85–94%) [45] (Figure 6).

The pooled intention to get the booster dose among HCWs was 77% (95% CI: 67–83%, *I*^2^ = 99%), while the pooled estimation of actual booster dose vaccination was 69% (95% CI: 56–79%, *p* = 0.080, Supplementary Figure S1).

The meta-regression for the pooled proportion of COVID-19 vaccine booster dose acceptance among HCWs, including the actual vaccination and intention to be vaccinated, explained 52.47% of the result. This meta-regression revealed that previous COVID-19 infection, large sample size greater than 1000 participants, and high-income country as the study setting had a significant effect on booster dose acceptance among the HCWs: 0.001 (95% CI: 0.001–0.003, *p* = 0.023), −1.23 (95% CI: −2.02 to −0.44, *p* = 0.002), and −1.75 (95% CI: −2.98 to −0.52, *p* = 0.005), respectively. A study setting in the Americas, Eastern Mediterranean region, or Western Pacific region had a statistically significant effect on booster dose acceptance among HCWs: 3.86 (95% CI: 1.80–5.91, *p* < 0.001), 4.19 (95% CI: 2.28–6.09, *p* < 0.001), and 3.48 (95% CI: 1.79–5.17, *p* < 0.001), respectively. The European region as a study region, being fully vaccinated, and employment had no statistically significant effect on booster dose acceptance among HCWs: 1.95 (95% CI: −0.05 to 3.23, *p =* 0.050), 0.54 (95% CI: −0.03 to 1.13, *p* = 0.065), and 0.31 (95% CI: −0.67 to 1.29, *p*= 0.533), respectively.

### 3.5. Acceptance of COVID-19 Booster Dose Vaccination across the WHO Regions

**Figure 7:**
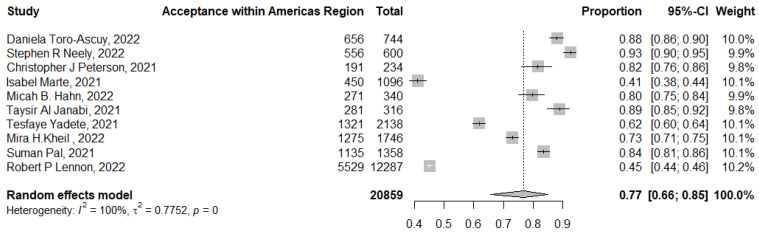
Acceptance of COVID-19 booster dose vaccination in the WHO region of the Americas. *p* = 0 denoted a *p*-value < 0.001.

The pooled acceptance of booster COVID-19 vaccination in the Americas Region, which did not include any actual uptake of booster doses, was 77% (95% CI: 66–85%, *I*^2^ = 100%), ranging from 93% (95% CI: 90–95%) [68] to 41% (95% CI: 38–44%) [46] (Figure 7).

**Figure 8:**
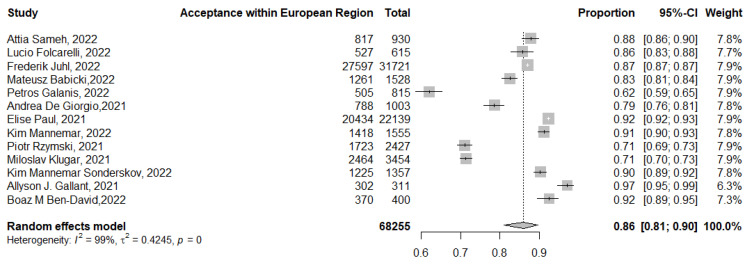
Acceptance of COVID-19 booster dose vaccination in the WHO region of Europe. *p* = 0 denoted a *p*-value < 0.001.

The pooled acceptance of booster dose COVID-19 vaccination in the European region was 86% (95% CI: 81–90%, *I*^2^ = 99%), ranging from 97% (95% CI: 95–99%) [37] to 62% (95% CI: 59–65%) [28] (Figure 8).

The subgroup analysis for the European region revealed that actual vaccination was 25% (95% CI: 10–48%, *I*^2^ = 99%), while the intention to receive the booster dose in the European region was 79% (95% CI: 65–88%, *I*^2^ = 100%).

**Figure 9:**
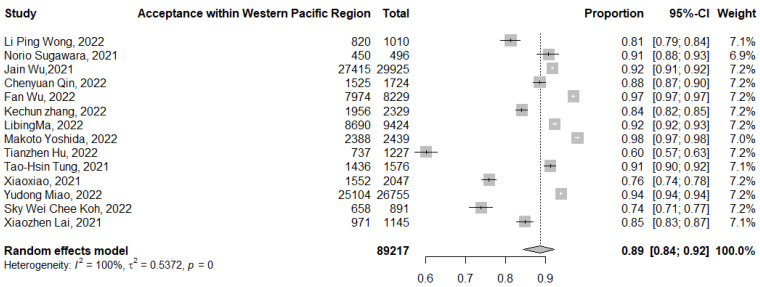
Acceptance of COVID-19 booster dose vaccination in the WHO Western Pacific region. *p* = 0 denoted a *p*-value < 0.001.

The pooled acceptance of the COVID-19 booster dose of the vaccine in the Western Pacific region was 89% (95% CI: 84–92%, *I*^2^ = 100%), ranging from 94% [69] to 60% [48] (Figure 9). The subgroup analysis for the Western Pacific region revealed that the pooled actual booster vaccination was 74% (95% CI: 71–77%) in a single study (Supplementary Figure S1) [56].

**Figure 10:**
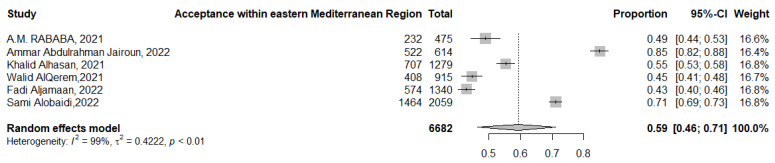
Acceptance of COVID-19 booster dose vaccination in the Eastern Mediterranean region.

The acceptance of booster COVID-19 vaccination in the Eastern Mediterranean region was 59% (95% CI: 46–71%, *I*^2^ = 99%), ranging from 71% (95% CI: 69–73%) [81] to 43% (95% CI: 40–46%) [62] (Figure 10).

The subgroup analysis revealed that the uptake of booster doses in the Eastern Mediterranean region was reported in one study [62].

The acceptance of booster COVID-19 vaccination in the Southeast Asian region was 52% (95% CI: 43–61%, *I*^2^ = 95%), and the subgroup revealed that the actual vaccination was 28% (95% CI: 8–66%), while the intention to have the booster dose, as reported in a single study, was 41% (95% CI: 39–43%). A single study reported the actual and intentional acceptance of the booster dose of COVID-19 vaccination in the African region (Supplementary Figure S1).

### 3.6. Predictors of COVID-19 Booster Dose Acceptance

**Figure 11:**
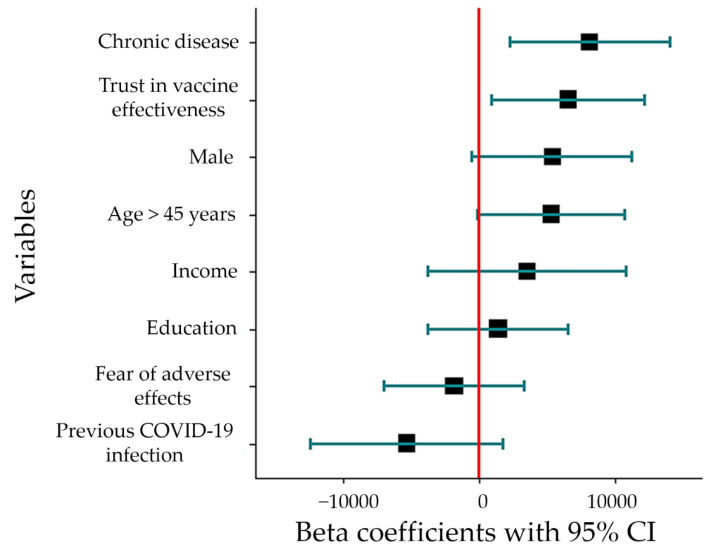
The predictors of COVID-19 vaccine booster dose acceptance.

Thirty-six of the 50 extracted studies discussed different predictors for COVID-19 vaccine booster dose acceptance. Age above 45 and the male gender were strong predictors detected in [41,54,60,61,69,74,78,81]. Educational level was a strong predictor as well, according to several included studies [28,37,45,52,61,65,68,69,70,72,76]. Being a HCW was another predictor in 2 studies [48,74], while a previous COVID-19 infection in the family was a predictor in 5 studies [28,49,60,61,63]. Employment status and personal/household income were predictors among several studies [47,51,56,61,72,76,81]. Having a history of chronic disease has been reported to be a predictor of booster dose acceptance in several studies [28,37,39,49,51,69,79], while other studies reported trust in the effectiveness of the vaccine and the fear of an unknown adverse effect as significant determinants of booster dose acceptance [35,37,42,51,66,67,70,75,77,78,79]. Among different populations, history of chronic disease and trust in the vaccine effectiveness were significant predictors through our linear regression model, which explained 39% of the predictors involved in COVID-19 booster dose acceptance: 8151 (95% CI: 2236–14064, *p* = 0.008) and 6548 (95% CI: 935–12159, *p* = 0.023, Figure 11).

## 4. Discussion

Effective and safe vaccines are considered critical in combating the COVID-19 pandemic by achieving population immunity that hinders virus spread [6,82,83,84]. Despite accumulated evidence showing the safety and effectiveness of the currently approved COVID-19 vaccines, the success of vaccination campaigns was challenged by the conspicuous barrier of COVID-19 vaccination hesitancy [8,9]. Several factors were shown to be correlated with lower acceptance of COVID-19 vaccination, including the sociodemographic characteristics and psychological factors, including (1) low confidence in vaccine safety and efficacy; (2) high complacency manifested in a higher perception of disease risks; (3) low convenience in terms of accessibility to vaccination services; (4) high calculation of the benefits and risks of vaccination; (5) low collective responsibility needed to protect the vulnerable groups in societies; and (6) high embrace of vaccine conspiracy beliefs [85,86,87,88].

Accordingly, we conducted a systematic review and meta-analysis to determine the approximate rate of vaccine hesitancy towards getting a booster dose of COVID-19 vaccination and to determine its associated factors. In turn, this can help to devise proper and well-informed intervention measures to improve vaccine acceptance, considering growing evidence that booster COVID-19 vaccination is necessary to control the pandemic [14,89]. This comes in light of the emergence of SARS-CoV-2 variants with immune escape potential besides the waning of population immunity [90,91,92].

In this meta-analysis, we aimed to assess the proportion of both the actual uptake of the COVID-19 vaccine booster dose and the intention to get the booster dose across the globe. The overall acceptance rate of booster COVID-19 vaccination among 198,831 subjects across 48 studies conducted in 23 countries was 81% (95% CI: 75–85%). This rate was higher compared with the recent and earlier estimates of COVID-19 vaccine acceptance, which ranged from 60% to 75% in various meta-analyses [85,93,94]. This higher estimated proportion of accepting the booster dose of COVID-19 vaccination can be related to the timing of the included studies, which were conducted in a recent time period compared with earlier studies tackling COVID-19 vaccine acceptance. In turn, this could have resulted in a more positive attitude towards vaccination, considering growing evidence of the safety and efficacy of the currently approved COVID-19 vaccines, highlighting the time specificity as an attribute of vaccination hesitancy [84,95,96].

The intention to accept a COVID-19 vaccine booster dose as estimated in this review was 79% (95% CI: 72–85%), while the actual booster dose vaccine uptake was 31% (95% CI: 19–46%). This observed disparity can also be linked to the timing of the studies included, where booster vaccination was not widely available, as well as the prioritization of high-risk groups. In this review, the acceptance of a booster dose among HCWs was 66% (95% CI: 58–74%), which is in line with the previous pooled estimates among health professionals worldwide [9,97]. Furthermore, this study confirmed the previous observation of regional differences in booster dose vaccine acceptance, consistent with previous reviews highlighting this issue [8,9]. It is known that vaccination hesitancy is place- and context-specific phenomenon; therefore, it is necessary to take into account these peculiarities in efforts aiming to promote COVID-19 vaccination [98].

In this review, the pooled acceptance of booster dose vaccination in the Americas, which did not include any actual uptake of booster vaccination, was 77% (95% CI: 66–85%). Higher rates of booster dose acceptance were reported in the Western Pacific (89%, 95% CI: 84–92%) and in the European region (86%, 95% CI: 81–90%). On the other hand, the lowest rates were reported in the Eastern Mediterranean region (59%, 95% CI: 46–71%) and the Southeast Asian region (52%, 95% CI: 43–61%). Thus, the high rates of COVID-19 vaccine hesitancy in the Middle East, which was shown previously, extended to involve hesitancy to booster doses as well [8,9,87].

The regional variability in COVID-19 booster dose vaccination can be attributed to the issues of vaccine equity and the implementation of different vaccine mandates [99]. Several low- and middle-income countries had struggles in relation to vaccine supplies, ending up in struggles to reach the intended goals of primary COVID-19 vaccination series [100]. On the other hand, a few high-income countries issued vaccine mandates in relation to COVID-19 booster dose vaccination with vaccine hoarding and low vaccine supply in other regions [101]. Prioritizing vaccine equity for the primary COVID-19 vaccination series should be considered to decrease the likelihood of SARS-CoV-2 variant emergence, which could be a major challenge to control the pandemic besides the issue of vaccination hesitancy [102].

A noteworthy finding of the current review was the scarcity of reports addressing COVID-19 booster dose vaccine acceptance in the African region. Besides the issue of vaccine equity and vaccination hesitancy that hinder the successful implementation of vaccination campaigns in the continents, lack of studies can be considered another obstacle that should be addressed urgently [8,103,104].

It was worthy to note that the overall acceptance rate of booster doses of COVID-19 vaccination was relatively high. However, the actual acceptance rate was relatively below the intentional acceptance rates. A potential explanation of this high acceptance rate is perceived safety of the currently available vaccines and perceived severity of COVID-19. In addition, the increase in trust in health authorities over the world can effectively affect the acceptance of vaccination [105]. A recent study that investigated COVID-19 booster dose vaccine acceptance in 14 East Mediterranean region countries showed that hesitancy to receive a booster dose was linked to concerns regarding the safety and efficacy of current vaccines [106]. The study also showed that low perceived benefit was a major determinant of the reluctance to have a booster dose of the COVID-19 vaccine [106].

To the best of our knowledge, our review is among the earliest and largest reviews to systematically assess COVID-19 vaccine booster dose acceptance at this scale involving the general public and health professionals; thus, we compared our findings with meta-analyses on vaccine acceptance. Our findings were higher than the results of previously published meta-analysis by Norhayati et al., which reported a pooled proportion of COVID-19 vaccine acceptance from 170 studies in 50 countries of 61% (95% CI: 59–64%) [93]. A recently published systematic review and meta-analysis by Galanis et al. estimated the acceptance of a COVID-19 vaccine booster dose at a level of 79%—similar to our estimate—among the general public based on the inclusion of 14 studies [107].

Interestingly, the acceptance rate of booster dose vaccination among HCWs was relatively lower than the average acceptance estimate. About one-third of HCWs were reluctant to receive booster doses. This finding is very crucial as HCWs play a key role in guiding local communities’ attitudes toward vaccination [108]. In addition, HCWs’ vaccination beliefs and attitudes are critical for primary prevention strategies [109,110]. However, this estimate should be interpreted in light of the relatively low number of studies and HCW participants compared with the general public. Therefore, this pattern is pending further studies to reach reliable conclusions about the attitude of HCWs towards booster dose COVID-19 vaccination.

In a recent study by Dziedzic et al., nearly three-quarters of those polled preferred receiving COVID-19 vaccine booster doses, while 17.6% and 7.9% expressed rejection and uncertainty, respectively [108]. In the previous study conducted in Poland, the authors speculated that the observed high acceptance rate of booster doses among HCWs may be due to the high level of health literacy [108]. Likewise, approximately 71.1% of Saudi HCWs indicated a willingness to receive a COVID-19 booster dose [81]. Thus, more studies are needed to confirm the finding of lower acceptance of booster dose vaccination among HCWs as observed in the current review.

In this study, the main identified predictors of booster dose acceptance were trust in vaccine effectiveness and the presence of a chronic disease among participants with increased vaccine acceptance linked to a history of a chronic disease. Such a result can be attributed to low levels of complacency among individuals with a chronic disease with high levels of perceived severity of COVID-19. Predictors such as age, gender, and fear of unknown adverse effects were insignificant predictors of booster dose acceptance.

In the context of the included studies, having a chronic illness increased the odds ratio of booster dose vaccine acceptance by 1.4 in the Algerian population [54]. Likewise, HCWs with chronic diseases opted more for booster doses of vaccination [81]. This may be related to higher levels of perceived severity and perceived benefit of vaccination compared with the normal population. The intention to receive a booster dose was significantly associated with nationality, marital status, gender, education level, monthly income, and comorbid medical illness. However, this high rate of booster dose vaccine acceptance was not observed in low-middle-income countries. This may be due to the steady increase in COVID-19 vaccine coverage in low- and middle-income countries; however, vaccine coverage in these countries remains lower than the rates reported in higher-income countries. 

The acceptance rate of the COVID-19 vaccine across WHO regions varied significantly, being the highest in the Western Pacific and the lowest in the Southeast Asian region. The pooled proportion of COVID-19 vaccine acceptance ranged across WHO regions from 52% in Southwest Asia to 89% in the Western Pacific. On the other hand, Norhayati et al. found that the pooled proportion of COVID-19 vaccine acceptance was the highest in Southeast Asia at 74% and the lowest in the Eastern Mediterranean region at 52% [93], which was consistent with an earlier review, which found that COVID-19 vaccine acceptance was over 90% in Southeast Asia, with the lowest proportions of acceptance in the Middle Eastern countries [9]. The Middle East’s low vaccine acceptance was linked to the widespread belief in conspiracies regarding emerging virus infections and subsequent control measures that harmed vaccination acceptance and uptake [87,111,112]. Furthermore, these variations may reflect varying levels of trust in information from government sources. Thus, cultural and regional aspects of vaccine hesitancy should be considered in intervention efforts needed to promote booster dose vaccine acceptance.

### Strengths and Limitations

The strengths of this review besides being the largest with such an aim and wide scope included: (1) the search was not limited to articles published in English, which may have allowed the generalizability of the review results. In addition, (2) we included studies with satisfactory, good, and very good quality of data based on the assessment of the risk of bias. Additionally, (3) we included preprints to increase the power of our study. Finally, (4) we searched many databases to find most, and possibly all, published studies addressing the acceptance and uptake of a booster dose of COVID-19 vaccines. The main limitation was that most of the records included in this review were cross-sectional studies, which can be thought of as snapshots of vaccine hesitancy status in each country/region. The included studies had different sampling strategies, variable survey instruments, and different assessment tools, which may explain some of the differences in vaccine acceptance rates reported in different studies from the same country. As a result, the findings should be regarded with caution, as these results cannot forecast future changes in vaccine acceptance rates.

## 5. Conclusions

The global acceptance rate of COVID-19 booster dose vaccination was found to be relatively high; however, the intention to have a booster dose was higher compared with the actual uptake of the booster dose. The relatively low acceptance rate of booster doses among HCWs is an alarming finding that should be studied in future studies. There is an observed difference in booster dose acceptance rates across WHO regions, which may shed light on the issue of vaccine inequity, besides possible links to cultural and regional differences in vaccine acceptance. To sum up, in order to achieve herd immunity against COVID-19, a high level of vaccination acceptance is required. Many vaccination campaigns and programs are still needed around the world to raise public awareness and acceptance of COVID-19 vaccines, including booster doses. These campaigns should consider the issues of effective coordination, engaging the public, and focusing on the safety and efficacy of the currently available vaccines [113,114]. In addition, policymakers should consider the importance of delivering concise messages highlighting the importance of booster dose vaccination needed to prevent the resurgence of COVID-19 cases and to protect vulnerable groups in the population [115,116].

## Figures and Tables

**Table 1:** Characteristics of studies included in the systematic review.

Study, Country	Design, Inclusion/Exclusion Criteria	Sample Size (*n*), Female (%), Age	Study Period	Previous COVID-19 (*n*)	Education/Employment	COVID-19 Vaccine Coverage (%)	Vaccine Type
Petersonet et al., USA [43]	Cross-sectional, medical students	234, 63%, NA	13 July 2021–3 August 2021	31	Medical students	Fully vaccinated (99.3)	Moderna and Pfizer
Sønderskov et al., Denmark [44]	Cross-sectional, those who received or were scheduled to receive vaccine and did not receive the booster dose were included; those who did not receive COVID-19 vaccine at all or received a booster dose were excluded	1357, NA, NA	10 December 2021–23 December 2021	NA	NA	Fully vaccinated (95.0)	Moderna and Pfizer
Wu et al., China [45]	Cross-sectional, individuals who are at least 18 years old and were able to read and complete the self-administered questionnaire independently were included	8229, 69%, 79% aged 26–45 years	24 October 2021–28 October 2021	24	Junior high school or below (33%), high school (29%), bachelor (36%), master, or above (2%). Employment as HCW (3%), other (98%)	Fully vaccinated (100)	NA
Marte et al., Dominican Republic [46]	Cross-sectional, all permanent residents over 18 years of age	1096, 60%, mean age: 37 years	July 2021	276	Bachelor (32%), master (28%), doctorate (21%), PhD (2%), technicians (7%), high school (11%), elementary school (0.3%); employment: public, private sector, or independently employed (85%), unemployed (15%)	Fully vaccinated (81.9)	Sinovac (68%), AstraZeneca (20%), Pfizer (4%), others (8%)
Al-Qerem et al., Jordan [47]	Cross-sectional, people aged 18 or above, living in Jordan, and fully vaccinated	915, NA, 46% were 18–29 years	1 October 2021–15 December 2021	NA	High school or less (7%), diploma (5%), university student (18%), bachelor (64%), postgraduates (6%)	Fully vaccinated (100)	Pfizer (57%), AstraZeneca (5%), Sinopharm (38%)
Hu et al., China [48]	Cross-sectional, NA ^2^	1227, 49%, 75% were 18–39 years	20 October 2021–10 December 2021	NA	Bachelor’s degree (53%), postgraduate and above (32%); employment: civil servants (12%), staff in government-affiliated public institutions (14%), enterprise employees (56%), doctors (2%)	Fully vaccinated (100)	NA
Yadete et al., USA [25]	Cross-sectional, NA	2138, 50%, NA	14 July 2021–19 July 2021	NA	NA	NA	NA
Jairoun et al., UAE [49]	Cross-sectional, students and faculty staff of Ajman University, aged 18 years and above	614, 69%, 38% were 23–26 years	25 August 2021–20 October 2021	NA	Primary school/elementary (19%), secondary education (32%), diploma (26%), university degree (11%), postgraduates (12%). Employment in health sector workers (27%)	Fully vaccinated (77)	NA
Qin et al., China [50]	Cross-sectional, Chinese citizen, having child aged under 18 years old	1724, 50%, 30 years or below 47%	12 November 2021–19 November 2021	NA	Bachelor’s degree (77%)	NA	NA
Babicki and Mastalerz-Migas. Poland [51]	Cross-sectional, over the age of 18, Poland resident, fully vaccinated, exclude no vaccination or incomplete vaccination	1528, 83%, NA	23 September 2021–3 October 2021	NA	University degree (78%)	Fully vaccinated (100)	Comirnaty, Spikevax, AstraZeneca, Johnson & Johnson, Pfizer
Zhang et al., China [52]	Cross-sectional, 18 years or above, a full-time employee of a factory in Shenzhen. Those who did not complete the primary vaccination series and those who received a booster dose were excluded	2329/ 51%, 46% were 30–39 years	26 October 2021–31 October 2021	NA	College/university or higher (46%), employment of a factory in Shenzhen	Fully vaccinated (93), partially vaccinated (7)	Sinopharm, Sinovac, CoronaVac, CanSino
Gallant et al., UK [37]	Longitudinal, adults aged 65 and older, living in the UK, independently in the community, were generally in good health	311, 48%, mean age: 70 years	February 2021–March 2021	NA	NA	Fully vaccinated (2), partially vaccinated (97)	NA
Iguacel et al., Colombia, El Salvador, and Spain [36]	Cross-sectional, 18 years or older, can read and complete the self-administered questionnaire independently	3026, 67%, 40% were 18–25 years	August 2021–December 2021	NA	High level (51%); employment: nurse (9%), medical doctor (7%), other health professionals (4%)	Fully vaccinated (78)	NA
Sønderskov et al., Denmark [38]	Longitudinal, adult population of Denmark	1555, 49%, mean age: 53 years	30 August 2021–15 September 2021	NA	Primary and lower secondary school (14%), upper secondary education (7%), vocational education (41%), short-cycle higher education (8%), medium-cycle higher education (21%), long-cycle higher education (10%)	Fully vaccinated or planned (95)	Pfizer, Moderna, Johnson, AstraZeneca, an combination
Alhasan et al., KSA ^1^ [53]	Cross-sectional, HCW ^3^	1279, 62%, mean age: 39 years	9–14 August 2021	297	Low level (23%), medium level (26%), high level (51%); employment: consultant (25%), assistant consultant/fellow (7%), resident/registrar/physician in training (19%), nurse (42%)	Fully vaccinated (69)	Pfizer or AstraZeneca
Lounis et al., Algeria [54]	Cross-sectional, Algerian national, at least 18 years old, capacity to communicate in Arabic or French, and being previously vaccinated against SARS-CoV-2	787, 62%, NA	28 January 2022–5 March 2022	514	Bachelor’s degree (44%), masters’ degree or above (48%); employment: HCW (39%), non-HCW (61%)	Fully vaccinated (100)	Sinovac (66%), Sinopharm (5%), AstraZeneca (13%), Janssen (3%), Sputnik V (10%), Pfizer (0.5%)
Klugar et al., Czechia [55]	Cross-sectional, HCW	3454, 81%, NA	3 to 11 November 2021	1105	Medical professionals (30%), allied health professionals (70%)	Received a third dose (49), fully vaccinated (50), received only one dose (2)	Pfizer (91%)
Koh et al., Singapore [56]	Cross-sectional, HCW, temporary staff, pharmacy and diagnostics staff were excluded	891, 85%, NA	1 January 2021–10 December 2021	NA	Administrative staff (14%), allied health workers (5%), ancillary services staff (37%), medical staff (19%), nursing staff (26%)	Fully vaccinated (99)	Pfizer, Moderna, CoronaVac, Sinopharm
Kheil et al., USA [57]	Cross-sectional, adults aged 18 years or older	1746, 55%, NA	18 October 2021–29 November 2021	NA	College (17%), bachelor’s degree (29%), master’s degree (15%), doctorate (25%)	Fully vaccinated or planned to receive the second dose (95)	Pfizer, Moderna, Johnson & Johnson
Wu et al., China [39]	Longitudinal study, Chinese adults, 18 years old or above	29,925, 51%, 18–39 years (84%)	6 to 9 August 2021	NA	High school graduate (26%), university graduate (61%)	NA	NA
Pal et al., USA [58]	Cross-sectional, adults aged 18 years and working in a healthcare setting in the US were included	1358, 79%, 31–60 years (71%)	1 February 2021–31 March 2021	924	Vocational (29%), bachelor’s degree (18%), master’s degree (12%), professional degree (28%); employment: DMP (40%), DPCP (38%), administration (10%)	Fully vaccinated or planning to receive both doses (92)	NA
Hahn et al., USA [40]	Longitudinal study, residents in remote Alaskan communities, aged 18 years or older; residents in Juneau were excluded	340, 70%, mean age: 43 years	9 November 2020–27 September 2021	NA	NA	Fully vaccinated (100)	NA
Yoshida et al., Japan [41]	Retrospective cohort study	2439, 58%, mean age: 53 years	December 2021	NA	NA	Fully vaccinated (100)	NA
Toro-Ascuy et al., Chile [59]	Cross-sectional, Chilean adult population, 18 years or older	744, 65%/ 18–59 years (95%)	May 2021–June 2021	NA	High school (37%), undergraduate (42%), postgraduate (21%)	Not vaccinated (100)	NA
Folcarelli et al., Italy [60]	Cross-sectional, fully vaccinated individuals in Naples and did not receive the booster dose	615, (57%), mean age: 32 years	November 2021–December 2021	102	High school or less (69%), bachelor/graduate degree (31%); employment: student (71%)	Fully vaccinated (100)	Pfizer
Wirawan et al., Indonesia [61]	Cross-sectional, residents of Jakarta and Bali, aged 18 years old, and had received at least one dose of the vaccine	2674, (58%), median age: 29 years	February 2022	62	Completed high school (53%), completed college (39%); employment: unemployed (13%), housewife (25%), student (12%), part-time employment (18%), full-time employment (33%)	NA	NA
Aljamaan et al., KSA [62]	Cross-sectional, parents who were residents in KSA	1340, 65%, 35–44 years (47%)	December 2021–January 2022	NA	University degree (76%); employment: unemployed/retired (22%), HCW (23%), employee (47%)	Fully vaccinated (61), booster (35)	NA
Wong et al., Malaysia [63]	Cross-sectional, fully vaccinated Malaysian residents aged 18 years or older	1010, 64%, mean age: 32 years	22 November 2021–9 February 2022	145	Professional and managerial (38%), general worker (14%), self-employed (6%), student (31%), housewife/retired/unemployed (11%)	Fully vaccinated (100)	NA
Rababa’h et al., Jordan [64]	Cross-sectional, Jordanian adults aged 18 and above	475, 76%, 18–39 years (75%)	August 2021	237	Bachelor (51%), graduate studies (34%); employed (58%), unemployed (38%)	NA	NA
Al Janabi and Pino. USA [35]	Cross-sectional, medical students	319, 51%, age range 18–49 years	Spring 2021	NA	NA	Full	NA
Paul and Fancourt. UK [65]	Cross-sectional	22,139, NA, NA	21 March 2020–6 December 2021	NA	NA	NA	NA
Mori et al., Japan [34]	Cross-sectional, medical staff at Sakaide	260, 74%, mean age: 40 years	2 December to 8 December 2021	NA	Medical doctors (13%), nurses (51%), administrative staff (24%)	NA	NA
Attia et al., Germany [66]	Cross-sectional, students and employees in German universities	930, 73%, mean age: 29 years	7 to 19 December 2021	55	322 were employees and 608 were students	NA	Pfizer was the most common
Lai et al., China [67]	Cross-sectional, Chinese adults	1145, 50%, age range: 18–59 years	June 2021	NA	College/associate/bachelor’s degree or above (73%), employed (87%)	Vaccinated (79%)	NA
Neely and Scacco. USA [68]	Cross-sectional	600, 52%, NA	July 2021	NA	NA	NA	NA
Motta. USA [42]	A retrospective observational study involving adults older than 18 years	1551, 54%, mean age: 46 years	22 to 27 April 2022	NA	NA	Fully vaccinated (72–78%)	NA
Miao et al., China [69]	Cross-sectional, residents in China, 18 years of older vaccinated individuals	26,755, 53%, NA	6 to 9 August 2021	NA	University graduate (63%)	Fully vaccinated	NA
Kunno et al., Thailand [70]	Cross-sectional, 18 years or older living in Bangkok and received the first dose of vaccination	780, 76%, mean age: 42 years	September 2021–December 2021	362	Bachelor’s (61%)	(97)	NA
Al Janabi and Pino. USA [71]	Cross-sectional, students at New York Institute of Technology College of Osteopathic Medicine (NYITCOM)	316, 47%, NA	Spring of 2021	NA	NA	Fully vaccinated (95)	Pfizer (61%), Moderna (34%), Janssen (5%)
Lennon et al., USA [72]	Cross-sectional	12,287, 51%, age range: 35 to 59 years old	7 May 2021–7 June 2021	NA	Some college, not graduate (30%), college graduate/postgraduate degree (30%)	Full	NA
Ben-David et al., Israel [73]	Cross-sectional	400/ 49%, mean age: 69 years	August 2021	NA	Academic education (53%)	NA	NA
Wang et al., China [74]	Cross-sectional, vaccinated Chinese adults were included	2047, NA, age range: 35–40 years	April to May 2021	NA	NA	(100)	NA
Tung et al., China [75]	Cross-sectional	1576, 77%, age: ≥40 years (53%)	August 2021	NA	Senior secondary school and below (49%), university and above 798 (51%)	Fully vaccinated (96)	NA
De Giorgio et al., Croatia [76]	Cross-sectional	1003, NA, NA	December 2021	NA	NA	Fully vaccinated (33)	Pfizer, AstraZeneca, Johnson & Johnson, Moderna
Rzymski et al., Poland [77]	Cross-sectional, included Polish aged 18 years or older and fully vaccinated	2427, 51%, age: <50 (62%)	September 2021	510	Tertiary education (71%)	(100)	Pfizer, others
Jørgensen et al., Denmark [78]	Cross-sectional, Danish citizens aged 18 or older	31,721, NA, NA	December 2021–13 February 2022	NA	NA	NA	Pfizer and Moderna
Ma et al., China [79]	Cross-sectional, included guardians of children aged <6 years in China	9424, NA, NA	15 September 2021–8 October 2021	NA	NA	NA	NA
Sugawara et al., Japan [80]	Cross-sectional, included medical students at Tokyo Medical University	496, 41%, mean age: 21 years	July 2021	NA	Medical students	(91)	NA
Alobaidi and Hashim. KSA [81]	Cross-sectional, HCWs in KSA aged >18 years	2059, 50%, mean age: 33 years	1 October 2021–30 November 2021	NA	NA	NA	NA
Galanis et al., Greece [28]	Cross-sectional, included those aged 18 years or above, had to understand the Greek language and fully vaccinated	815, 76%, mean age: 37 years	23 May to 30 May 2022	450	NA	(100)	NA

^1^ KSA: Kingdom of Saudi Arabia; ^2^ NA: not applicable or the information was not available.^3^ HCW: healthcare worker; USA: United states of America; UAE: United Arab of Emirates.

**Table 2:** Intention to receive booster COVID-19 vaccination and its predictors in the included studies.

Study, Country	Survey Tool Used	Valid Study Outcome Predictors	Participants Accepting Booster Dose Total	Actual	Intention	Study Quality Score
Peterson et al., USA [43]	Online	NA ^2^	191 (82%)	0	82%	Satisfactory
Sønderskov et al., Denmark [44]	Online	NA	1225 (95)	0	95%	Unsatisfactory
Wu et al., China [45]	Online	Gender, age, occupation, discomfort after vaccination, interval after last vaccination, active attention to news, PMT ^3^ scale (threat appraisal, response efficacy, self-efficacy, and response cost), VHS ^4^ scale (complacency, convenience, and confidence)	7974 (97)	0	97%	Satisfactory
Marte et al., Dominican Republic [46]	Online	NA	450 (41)	0	41%	Satisfactory
Al-Qerem et al., Jordan [47]	Online	Household average monthly income, severity of symptoms, deliberate receipt of COVID-19 vaccination status, risk level	408 (45)	0	45%	Satisfactory
Hu et al., China [48]	Online	NA	737 (60)	0	60%	Satisfactory
Yadete et al., USA [25]	Online	NA	1321 (62)	0	62%	Satisfactory
Jairoun et al., UAE [49]	Online	Employment, chronic disease status, having relatives infected with COVID-19, COVID-19 full vaccination status, admission to hospital due to COVID-19	522 (85)	0	85%	Satisfactory
Qin et al., China [50]	Online	NA	1525 (88)	0	88%	Satisfactory
Babicki and Mastalerz-Migas. Poland [51]	Online	Confidence level, age, chronic disease status, adverse event occurrence	1069 (69.9)	2.5%	67.4%	Satisfactory
Zhang et al., China [52]	Online	Social-media-related perceptions to booster dose, gender, educational level, monthly personal income, status as frontline workers or management staff	1956 (84)	0	84%	Satisfactory
Gallant et al., UK [37]	Online	NA	302 (97.1)	0	97.1%	Unsatisfactory
Iguacel et al., Colombia, El Salvador, and Spain [36]	Online	Age, gender, occupational status, and vaccination status	2403 (79.4)	0	79.4%	Unsatisfactory
Sønderskov et al., Denmark [38]	Online	Age	1418 (91.2)	0	91.2%	Satisfactory
Alhasan et al., KSA ^1^ [53]	Online	Nationality, full vaccination status, precaution implementation perception, awareness about the delta variant, and vaccination regimen effectiveness expectations	707 (55.3)	0	55.3%	Satisfactory
Lounis et al., Algeria [54]	Online	Age, sex, education, profession, COVID-19 infection status, postvaccination relief, postvaccination perceptions	406 (51.6)	0	51.6	Good
Klugar et al., Czechia [55]	Online	NA	2463 (71.3)	48.5%	22.8%	Very good
Koh et al., Singapore [56]	Record review	Sex, workplace, and profession as the key factor in affecting time to COVID-19 booster vaccination	658 (73.8)	73.8%	0	Very good
Kheil et al., USA [57]	Online	NA	1275 (73)	0	73%	Satisfactory
Wu et al., China [39]	Hybrid	Age, sex, educational level, marital status, chronic disease condition, smoking status	26,340 (88.02)	0	88.02%	Good
Pal et al., USA [58]	Online	NA	1135 (83.6)	0	83.6%	Satisfactory
Hahn et al., USA [40]	Online	NA	271 (79.7)	0	79.7	Satisfactory
Yoshida et al., Japan [41]	Face-to-face	Age, sex, number of adverse reactions after the second vaccination, antibody titer, and place of residence	2388 (97.9)	97.9%	0	Satisfactory
Toro-Ascuy et al., Chile [59]	Online	Trust in vaccine status, trust in stakeholders’ status, trust in social media status, trust in press status	656 (88.2)	0	88.2%	Good
Folcarelli et al., Italy [60]	Online	Age, gender, marital status, having cohabitants, education, COVID-19 infection status for the participant or his/her relevant or friends, COVID-19 booster awareness	527 (85.7)	85.7%	0	Good
Wirawan et al., Indonesia [61]	Online	Health beliefs, media influence, trust in authoritative sources, age, sex, religion, education level, employment status, monthly income, health insurance, and COVID-19 history	1505 (56.3)	15.1	41.2	Satisfactory
Aljamaan et al., KSA [62]	Online	NA	574 (42.8)	42.8%	0	Unsatisfactory
Wong et al., Malaysia [63]	Online	Age group, ethnicity, marital status, average monthly household income, region, past COVID-19 vaccination side effect status, severity of side effects after vaccination, pandemic fatigue status, practices of recommended measures against COVID-19 infection	820 (81.2)	0	81.2%	Good
Rababa’h et al., Jordan [64]	Online	Side effects status	232 (49)	0	49%	Satisfactory
Al Janabi and Pino. USA [35]	Online	Age, gender, marital status, race/ethnicity, household income, campus location, vaccine type	281 (88.9)	0	88.9%	Satisfactory
Paul and Fancourt. UK [65]	Online	Gender, age, ethnicity, education, smoking status, employment status, area of dwelling	-	0	92%	Satisfactory
Mori et al., Japan [34]	Online	Age, pregnancy status for females, side effect status	25 (93.1)	0	93.1%	Unsatisfactory
Attia et al., Germany [66]	Online	Gender, age, employment status, pregnancy status (for females), ethical opinion of vaccine justice, vaccine safety opinion	817 (87.8)	27.2%	60.6%	Good
Lai et al., China [67]	Online	Age, gender, maternal status, education level, employment status, household annual income, residence, and region	971 (84.8)	0	84.8%	Very good
Neely and Scacco. USA [68]	Online	Age, gender, political affiliation, ethnicity, residence, and region	556 (92.6)	0	92.6%	Good
Motta. USA [42]	Online	Age, gender, education level, employment status, respondents’ political ideology	760 (49)	49%	0	Good
Miao et al., China [69]	Online	Age, gender, educational status, ethnicity, religion, marital status, social level, chronic condition status, smoking status, washing hands status, wearing mask, gathering activities, COVID-19 conspiracy beliefs, risk of COVID-19 infection, curability of COVID-19, vaccine adverse reactions, channel of vaccine information, vaccine conspiracy beliefs, convenience, effectiveness, trust	25,105 (93.83)	0	93.83%	Satisfactory
Kunno et al., Thailand [70]	Online	Level of confidence in the effectiveness of the booster dose and the occurrence of adverse events in them or their loved ones, marital status, education level, occupation	366 (46.9)	46.9%	0	Satisfactory
Al Janabi and Pino. USA [71]	Online	Pharma mistrust, vaccine-induced immunity, vaccines adverse effects	224 (70.2)	70.2%	0	Unsatisfactory
Lennon et al., USA [72]	Mixed (phone and online)	Race, ethnicity, educational level, median income, party identification, geography/urbanity	5530 (45)	0	45%	Satisfactory
Ben-David et al., Israel [73]	Online	Academic education, contracting COVID-19, sense of control	370 (92.3)	60%	32.3%	Unsatisfactory
Wang et al., China [74]	Online	Age, gender, healthcare workers, high education	1552 (75.8)	0	75.8%	Satisfactory
Tung et al., China [75]	Online	History of allergic reaction, concerns about vaccine effectiveness and safety	1436 (91.1)	0	91.1%	Satisfactory
De Giorgio et al., Croatia [76]	Online	Unrealistic optimism, age, educational level, employment, loss of a close person, sources of information regarding COVID-19 and vaccines	789 (78.6)	0	78.6%	Satisfactory
Rzymski et al., Poland [77]	Online	COVID-19 vaccine-related side effects status, vaccine trust status	1724 (71)	0	71%	Satisfactory
Jørgensen et al., Denmark [78]	Online	Age, sex, societal threat, response efficacy, self-efficacy, response cost	27,598 (87)	0	87%	Satisfactory
Ma et al., China [79]	Online	Guardians’ education level, children disease status, guardians’ vaccination status, vaccine safety and effectiveness concern status	8690 (92.21)	0	92.21%	Satisfactory
Sugawara et al., Japan [80]	Online	Development of COVID-19 vaccines parents’ opinion	450 (90.7)	0	90.7%	good
Alobaidi and Hashim. KSA [81]	Online	Gender, age, nationality, marital status, educational level, monthly income, comorbid medical illness status, health beliefs	1464 (71.1)	0	71.1%	Unsatisfactory
Galanis et al., Greece [28]	Online	Educational level, comorbidity status, influenza vaccination status, self, relatives COVID-19 infection status	506 (62)	0	62%	Satisfactory

^1^ KSA: Kingdom of Saudi Arabia; ^2^ NA: not applicable or the information was not available; ^3^ PMT: protection motivation theory; ^4^ VHS: vaccine hesitancy scale; COVID-19: Coronavirus diseases 2019.

## Data Availability

The full data that support this systematic review are available in the included studies in the reference section. The analyzed data are available from the corresponding authors (M.S and R.M.G.) upon request. PROSPERO registration: CRD42022333758.

## Supplementary Materials

The following supporting information can be downloaded at: https://www.mdpi.com/article/10.3390/tropicalmed7100298/s1, Table S1: Search strategy; Figure S1: Additional results.

## Abbreviations

CI	confidence interval
COVID-19	coronavirus disease 2019
DOI	digital object identifier
EUL	emergency use listing
HCWs	healthcare workers
PRISMA	Preferred Reporting Items for Systematic Reviews and Meta-Analyses
QA	quality assessment
SARS-CoV-2	severe acute respiratory syndrome coronavirus 2
VIF	variance inflation factor
WHO	World Health Organization
