# Supplementary material for: COVID-19 Vaccine Booster Dose Acceptance: Systematic Review and Meta-Analysis
Source: Trop Med Infect Dis. 2022 Oct 13;7(10):298. doi: 10.3390/tropicalmed7100298 (PMC9611447; doi:10.3390/tropicalmed7100298)
Supplement: Supplementary file 1 [file tropicalmed-07-00298-s001.zip › Figure S1.pdf]

Figure S1: Additional Results

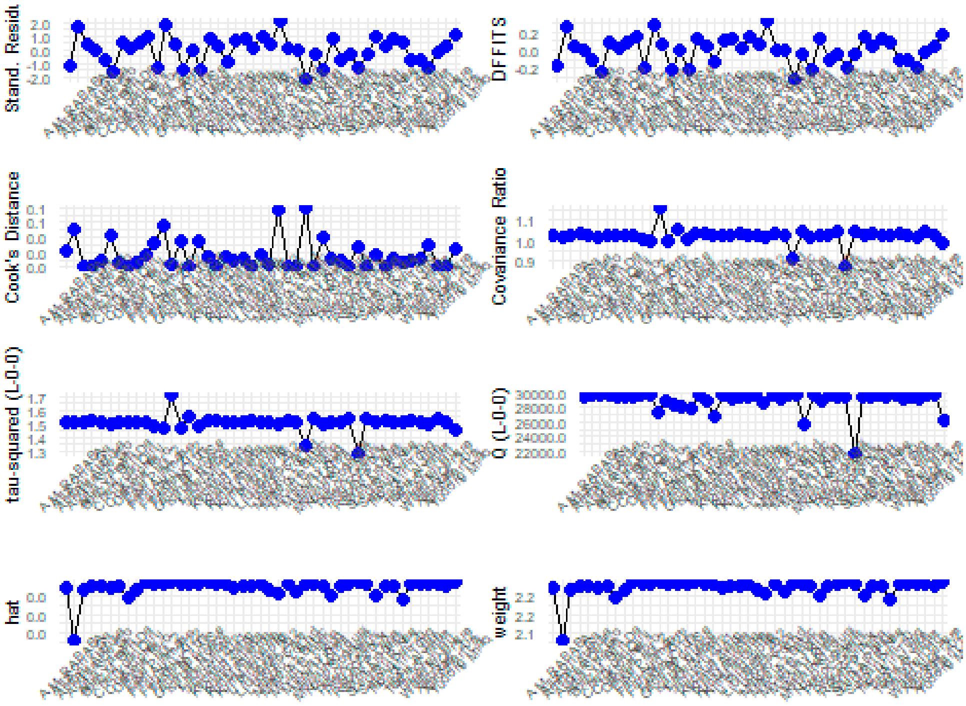

Leave one out analysis for intention to get the booster dose of COVID-19 vaccines

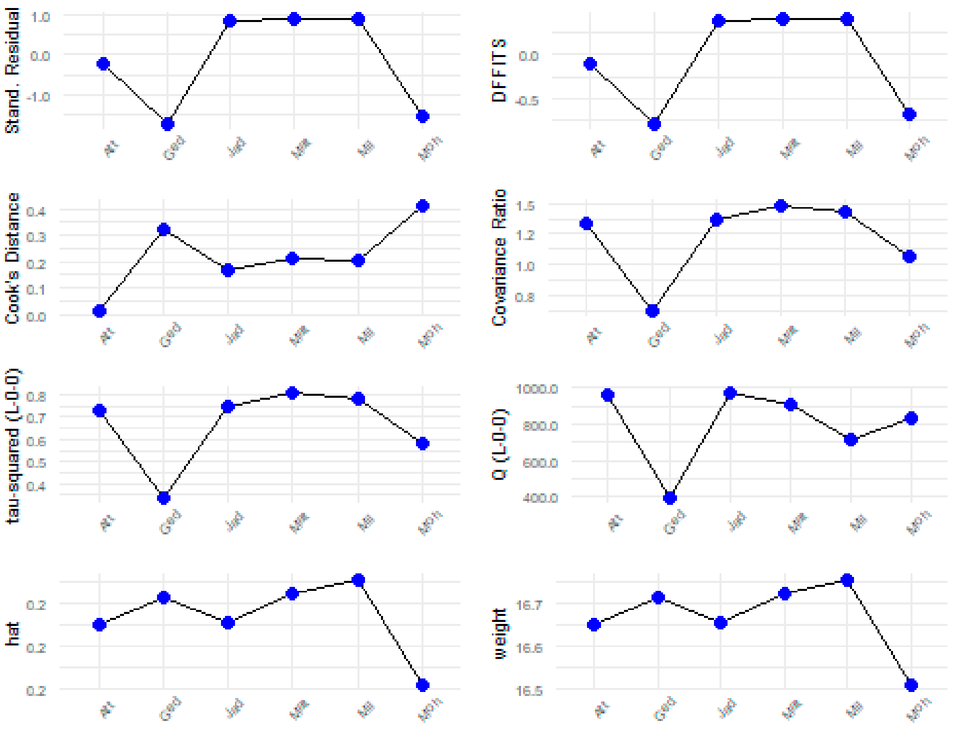

Leave one out analysis for the actual uptake of the COVID-19 vaccine booster dose

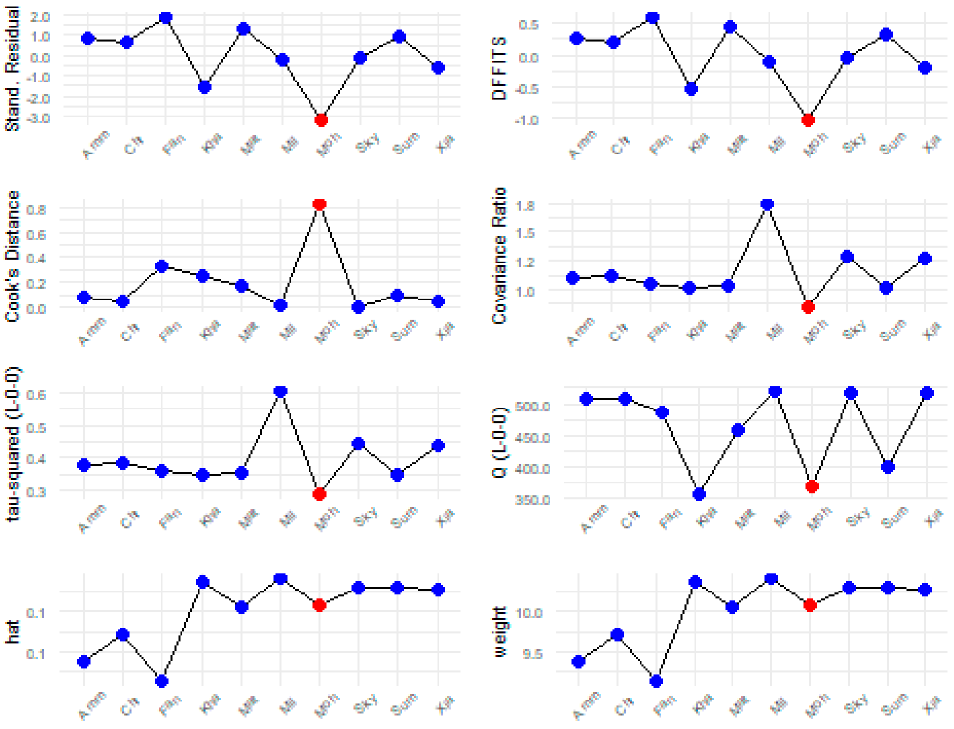

Leave one out analysis for HCWs acceptance of booster dose of COVID-19 vaccines

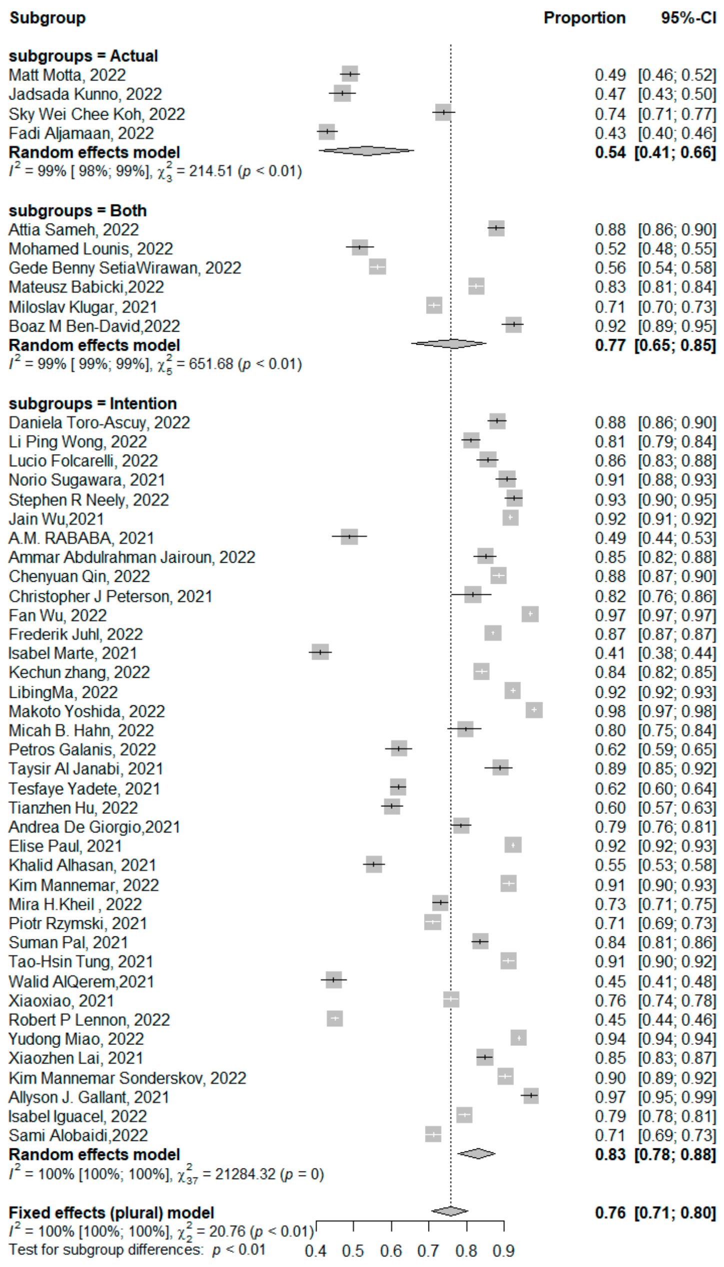

Subgroup analysis for total booster COVID-19 vaccine acceptance according to actual uptake and intention to get vaccinated

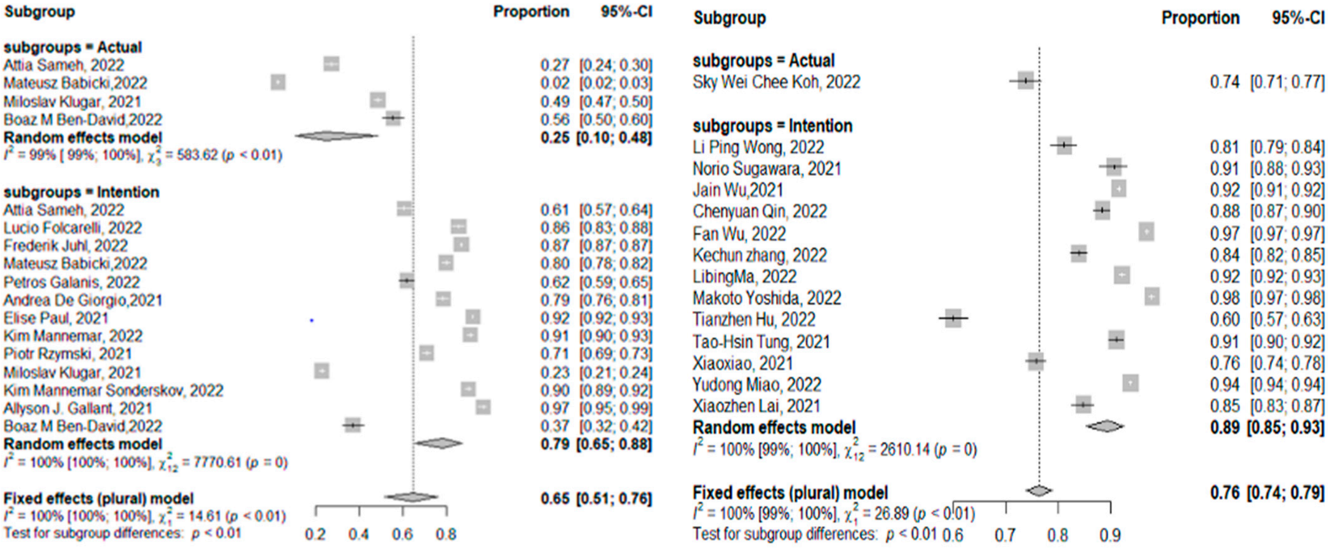

(A) **(B)**

Subgroup actual and intentional vaccination in the European region (A); and the Western Pacific region (B).

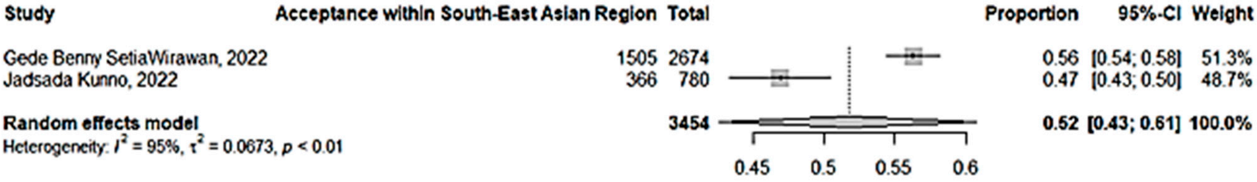

Booster dose COVID-19 vaccine acceptance in the South-East Asian region

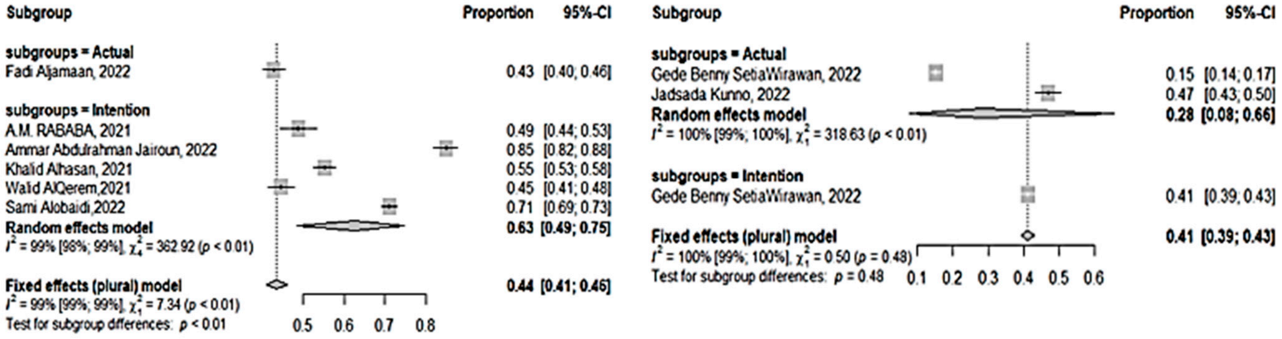

(A) **(B)**

Subgroup analysis of COVID-19 booster dose vaccine acceptance in the Eastern Mediterranean (A) and South-East Asian (B) regions

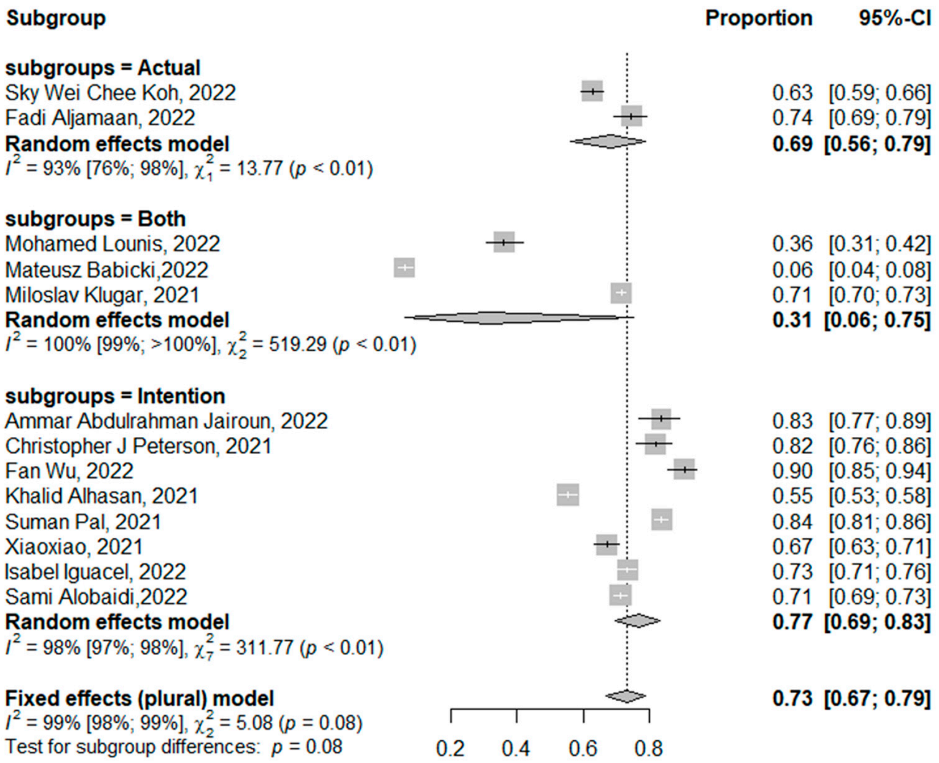

Subgroup analysis of healthcare workers (HCWs) actual and intention to accept the booster dose.
