# Supplementary material for: COVID-19 Vaccine Booster Dose Acceptance: Systematic Review and Meta-Analysis
Source: Trop Med Infect Dis. 2022 Oct 13;7(10):298. doi: 10.3390/tropicalmed7100298 (PMC9611447; doi:10.3390/tropicalmed7100298)
Supplement: Supplementary file 1 [file tropicalmed-07-00298-s001.zip › Table S1.pdf]

Table S1. Search strategy

Database	Keywords using MeSH and Boolean Operators	Number of citations
PsycINFO	(Coronavirus Disease 2019 Virus OR 2019 Novel Coronavirus OR 2019 Novel Coronaviruses OR SARS-CoV-2 Virus OR SARS-CoV-2 Virus OR SARS-CoV-2 Viruses OR 2019-nCoV OR COVID-19 Virus OR COVID 19 Virus OR COVID-19 Viruses OR SARS Coronavirus 2 OR Severe Acute Respiratory Syndrome Coronavirus 2 OR COVID 19 OR COVID-19 Virus Disease OR COVID 19 Virus Disease OR COVID-19 Virus Diseases OR COVID-19 Virus Infection OR COVID 19 Virus Infection OR COVID-19 Virus Infections OR 2019-nCoV Infection OR 2019 nCoV Infection OR 2019-nCoV Infections OR Coronavirus Disease-19 OR Coronavirus Disease 19 OR 2019 Novel Coronavirus Disease OR 2019 Novel Coronavirus Infection OR 2019-nCoV Disease OR 2019 nCoV Disease OR 2019-nCoV Diseases OR Coronavirus Disease 2019 OR SARS Coronavirus 2 Infection OR SARS-CoV-2 Infection OR SARS-CoV-2 Infection OR SARS-CoV-2 Infections) AND ("COVID-19 vaccine booster" OR "COVID-19 booster shot" OR "COVID-19 vaccine third dose" OR "COVID-19 vaccine additional dose") AND ("Hesitancy, Vaccination" OR "Vaccination Hesitancies" OR "Vaccine Hesitancy" OR "Hesitancy, Vaccine" OR "Vaccine Hesitancies" OR "Vaccine Delay" OR "Delay, Vaccine" OR "Delays, Vaccine" OR "Vaccine Delays" OR "Vaccination Delay" OR "Delay, Vaccination" OR "Delays, Vaccination" OR "Vaccination Delays")	100
Scopus	TITLE-ABS-KEY ((coronavirus OR "Middle East respiratory syndrome" OR "Severe acute respiratory syndrome" OR "Porcine epidemic diarrhea virus" OR "Feline infectious peritonitis virus" OR "Murine hepatitis virus" OR "Avian infectious bronchitis virus") AND ("cvid-19 vaccine booster" OR "cvid-19 booster shot" OR "cvid-19 vaccine third dose" OR "cvid-19 vaccine additional dose"))	35
EBSCO	(TI(coronavirus) OR TI(coronavirus infections) OR TI(coronavirus) OR TI(covid 2019) OR TI(SARS2) OR TI(SARS-CoV-2) OR TI(SARS-CoV-19) OR TI(severe acute respiratory syndrome coronavirus 2) OR TI(coronavirus infection) OR TI(severe acute	291

	respiratory pneumonia outbreak)OR TI(novel cov)OR TI(2019ncov) OR TI(sars cov2) OR TI(cov22) OR TI(ncov) OR TI(covid-19) OR TI(covid19) OR TI(coronaviridae) OR TI(corona virus)) AND ((TI=("COVID-19 vaccine booster" OR "COVID-19 booster shot" OR "COVID-19 vaccine third dose" OR "COVID-19 vaccine additional dose"))	
MEDLINE central/PubMed	(Coronavirus Disease 2019 Virus OR 2019 Novel Coronavirus OR 2019 Novel Coronaviruses OR SARS-CoV-2 Virus OR SARS-CoV-2 Virus OR SARS-CoV-2 Viruses OR 2019-nCoV OR COVID-19 Virus OR COVID 19 Virus OR COVID-19 Viruses OR SARS Coronavirus 2 OR Severe Acute Respiratory Syndrome Coronavirus 2 OR COVID 19 OR COVID-19 Virus Disease OR COVID 19 Virus Disease OR COVID-19 Virus Diseases OR COVID-19 Virus Infection OR COVID 19 Virus Infection OR COVID-19 Virus Infections OR 2019-nCoV Infection OR 2019 nCoV Infection OR 2019-nCoV Infections OR Coronavirus Disease-19 OR Coronavirus Disease 19 OR 2019 Novel Coronavirus Disease OR 2019 Novel Coronavirus Infection OR 2019-nCoV Disease OR 2019 nCoV Disease OR 2019-nCoV Diseases OR Coronavirus Disease 2019 OR SARS Coronavirus 2 Infection OR SARS-CoV-2 Infection OR SARS-CoV-2 Infection OR SARS-CoV-2 Infections) AND ("COVID-19 vaccine booster" OR "COVID-19 booster shot" OR "COVID-19 vaccine third dose" OR "COVID-19 vaccine additional dose") AND ("Hesitancy, Vaccination" OR "Vaccination Hesitancies" OR "Vaccine Hesitancy" OR "Hesitancy, Vaccine" OR "Vaccine Hesitancies" OR "Vaccine Delay" OR "Delay, Vaccine" OR "Delays, Vaccine" OR "Vaccine Delays" OR "Vaccination Delay" OR "Delay, Vaccination" OR "Delays, Vaccination" OR "Vaccination Delays")	PubMed 13<br>PubMed Central 73
ProQuest	(MAINSUBJECT.EXACT("Severe acute respiratory syndrome coronavirus 2") OR MAINSUBJECT.EXACT("COVID-19")) AND ("COVID-19 vaccine booster" OR "COVID-19 booster shot" OR "COVID-19 vaccine third dose" OR "COVID-19 vaccine additional dose") AND ("Hesitancy, Vaccination" OR "Vaccination Hesitancies" OR "Vaccine Hesitancy" OR "Hesitancy, Vaccine" OR "Vaccine Hesitancies" OR "Vaccine Delay" OR "Delay, Vaccine" OR "Delays,	52

	Vaccine" OR "Vaccine Delays" OR "Vaccination Delay" OR "Delay, Vaccination" OR "Delays, Vaccination" OR "Vaccination Delays" OR MAINSUBJECT.EXACT("Acceptance") OR MAINSUBJECT.EXACT("Social perception") OR MAINSUBJECT.EXACT("Attitudes") OR MAINSUBJECT.EXACT("Opinions") MAINSUBJECT.EXACT("Public opinion") OR MAINSUBJECT.EXACT("Perceptions") MAINSUBJECT.EXACT("Complaints"))	
SciELO	(Covid-19 OR SARS-Cov-2 OR SARS-Cov-19 OR coronavirus) AND (vaccine booster OR vaccine third dose OR additional dose) AND (acceptance) OR (hesitancy) OR (vaccine delay)	3
SAGE	for [[All "coronavirus disease 2019 virus"] OR [All "2019 novel coronavirus"] OR [All "2019 novel coronaviruses"] OR [All "sars-cov-2 virus"] OR [All "sars-cov-2 virus"] OR [All "sars-cov-2 viruses"] OR [All "2019-ncov or covid-19 virus"] OR [All "covid 19 virus"] OR [All "covid-19 viruses"] OR [All "sars coronavirus 2"] OR [All "severe acute respiratory syndrome coronavirus 2"] OR [All "covid 19"] OR [All "covid-19 virus disease"] OR [All "covid 19 virus disease"] OR [All 'covid-19]] AND [All virus diseases] AND [All " or "] AND [All covid-19 virus infection] AND [All " or "] AND [All covid 19 virus infection] AND [All " or "] AND [All covid-19 virus infections] AND [All " or "] AND [All 2019-ncov infection] AND [All " or "] AND [All 2019 ncov infection] AND [All " or "] AND [All 2019-ncov infections] AND [All " or "] AND [All coronavirus disease-19] AND [All " or "] AND [All coronavirus disease 19] AND [All " or "] AND [All 2019 novel coronavirus disease] AND [All " or "] AND [All 2019 novel coronavirus infection] AND [All " or "] AND [All 2019-ncov disease] AND [All " or "] AND [All 2019 ncov disease] AND [All " or "] AND [All 2019-ncov diseases] AND [All " or "] AND [All coronavirus disease 2019] AND [All " or "] AND [All sars coronavirus 2 infection] AND [All " or "] AND [All sars-cov-2 infection] AND [All " or "] AND [All sars-cov-2 infection] AND [All " or "] AND [All sars-cov-2 infections] AND [All ") and ("] AND [All covid-19 vaccine booster] AND [All " or "] AND	1

	[All covid-19 booster shot] AND [All " or "] AND [All covid-19 vaccine third dose] AND [All " or "] AND [All covid-19 vaccine additional dose] AND [All ") and ("] AND [All hesitancy, vaccination] AND [All " or "] AND [All vaccination hesitancies] AND [All " or "] AND [All vaccine hesitancy] AND [All " or "] AND [All hesitancy, vaccine] AND [All " or "] AND [All vaccine hesitancies] AND [All " or "] AND [All vaccine delay] AND [All " or "] AND [All delay, vaccine] AND [All " or "] AND [All delays, vaccine] AND [All " or "] AND [All vaccine delays] AND [All " or "] AND [All vaccination delay] AND [All " or "] AND [All delay, vaccination] AND [All " or "] AND [All delays, vaccination] AND [All " or "] AND [All vaccination delays"]	
Web of Science	(TI=("Wuhan coronavirus" OR "COVID19*" OR "COVID-19*" OR "COVID-2019*" OR "coronavirus disease 2019" OR "SARS-CoV-2" OR "2019-nCoV" OR "2019 novel coronavirus" OR "severe acute respiratory syndrome coronavirus 2" OR "2019 novel coronavirus infection" OR "coronavirus disease 2019" OR "coronavirus disease-19" OR "SARS-CoV-2019" OR "SARS-CoV-19")) AND (TI=("COVID-19 vaccine booster" OR "COVID-19 booster shot" OR "COVID-19 vaccine third dose" OR "COVID-19 vaccine additional dose")) AND (TI=("Hesitancy, Vaccination" OR "Vaccination Hesitancies" OR "Vaccine Hesitancy" OR "Hesitancy, Vaccine" OR "Vaccine Hesitancies" OR "Vaccine Delay" OR "Delay, Vaccine" OR "Delays, Vaccine" OR "Vaccine Delays" OR "Vaccination Delay" OR "Delay, Vaccination" OR "Delays, Vaccination" OR "Vaccination Delays"))	31
Google Scholar	“COVID-19 vaccine booster” OR “COVID-19 booster” OR “COVID-19 third dose” OR “COVID-19 additional dose” AND “Hesitancy” OR "Acceptance"	460
ScienceDirect	("coronavirus" OR " covid-19”) AND (" vaccine booster" OR "vaccine third dose" OR " vaccine additional dose") AND (" acceptance" OR "hesitancy")	20
